# Supplementary material for: Labour market marginalisation in young refugees and their majority peers in Denmark and Sweden: The role of common mental disorders and secondary school completion
Source: PLoS One. 2022 Feb 16;17(2):e0263450. doi: 10.1371/journal.pone.0263450 (PMC8849515; doi:10.1371/journal.pone.0263450)

S1 Figure. The proportion experiencing labour market marginalisation (LMM) from age 20-29 in refugees and the majority population in Denmark. Students identified using stipend data or enrolment data.

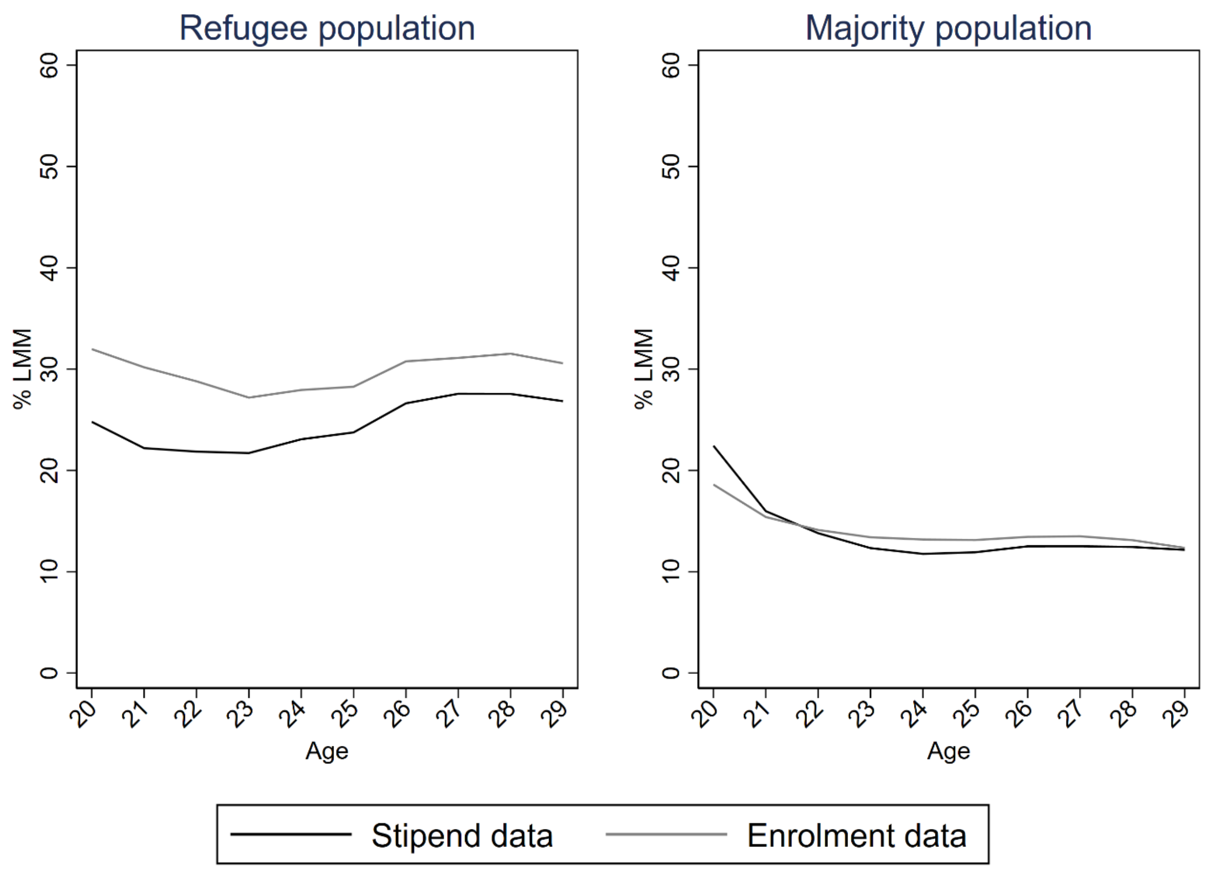

Supplement: S1 Fig — Students identified using stipend data or enrolment data. (PDF) [file pone.0263450.s001.pdf]
