# Supplementary material for: Labour market marginalisation in young refugees and their majority peers in Denmark and Sweden: The role of common mental disorders and secondary school completion
Source: PLoS One. 2022 Feb 16;17(2):e0263450. doi: 10.1371/journal.pone.0263450 (PMC8849515; doi:10.1371/journal.pone.0263450)
Supplement: S1 Table — Stratified by sex. (PDF) [file pone.0263450.s002.pdf]

S1 Table. The mediating role of secondary school completion and common mental disorders in the inequality in labour market marginalisation between young refugees and their majority peers. Stratified by sex.

	**DENMARK**				**SWEDEN**			
	Boys		Girls		Boys		Girls	
	OR_{men}	95% CI	OR_{women}	95% CI	OR_{men}	95% CI	OR_{women}	95% CI
**Model 1: Mediation by secondary school completion**								
Total effect	2.19	[2.09-2.29]	1.96	[1.85-2.07]	2.14	[2.08-2.20]	2.57	[2.49-2.64]
Controlled direct effect	1.32	[1.24-1.41]	1.11	[1.02-1.20]	1.11	[1.06-1.17]	1.40	[1.31-1.49]
Portion eliminated	1.65	[1.58-1.73]	1.76	[1.67-1.87]	1.93	[1.88-1.98]	1.84	[1.78-1.89]
Proportion eliminated (%)	73%		89%		90%		75%	
**Model 2: Mediation by common mental disorders**								
Total effect	2.19	[2.09-2.29]	1.96	[1.85-2.07]	2.14	[2.08-2.20]	2.57	[2.49-2.64]
Controlled direct effect	2.16	[2.05-2.27]	2.31	[2.17-2.46]	2.27	[2.21-2.33]	3.03	[2.94-3.13]
Portion eliminated	0.97	[0.94-1.00]	0.91	[0.87-0.94]	0.94	[0.92-0.97]	0.85	[0.82-0.87]
Proportion eliminated (%)			.				.	
**Model 3: Mediation by both**								
Total effect	2.19	[2.09-2.29]	1.96	[1.85-2.07]	2.14	[2.08-2.20]	2.57	[2.49-2.64]
Controlled direct effect	1.37	[1.28-1.46]	1.37	[1.26-1.49]	1.21	[1.15-1,28]	1.79	[1.67-1.90]
Portion eliminated	1.60	[1.52-1.67]	1.43	[1.35-1.51]	1.76	[1.72-1.81]	1.44	[1.39-1.48]
Proportion eliminated (%)	69%		61%		82%		50%	

Notes: Labour market marginalisation: labour market income of less than 12.5% of the median labour market income in the population aged 20-64 and not a student and is measured 2012-2016. Secondary school corresponds to a completed education at ISCED level 3 or above during the year prior to measurement of marginalisation (2011-2015) on 1 October in Denmark and 31 December in Sweden. Common mental disorders are psychiatric hospital contacts (inpatient or specialised outpatient) with a main diagnosis related to ICD-10 codes F32-33 or F40-43 or the purchase of a prescribed anti-depressant (ATC code N06A). Sex, birth year and municipality type were entered as covariates in the relationship between population group and mediator. Proportion eliminated omitted if the portion eliminated differs in direction from the controlled direct effect. Total effect corresponds to the overall inequality between the two groups.
