# Supplementary material for: Labour market marginalisation in young refugees and their majority peers in Denmark and Sweden: The role of common mental disorders and secondary school completion
Source: PLoS One. 2022 Feb 16;17(2):e0263450. doi: 10.1371/journal.pone.0263450 (PMC8849515; doi:10.1371/journal.pone.0263450)
Supplement: Attachment — Submitted filename: Response to reviewers.docx [file pone.0263450.s003.docx]

**Response to reviewers related to manuscript PONE-D-21-19639**

Dear Editor and Reviewer,

Thank you for taking the time to process and review our manuscript and for the comments provided. In the following, we respond to each comment.

**1. Rationale for using dichotomous indicators for labour market marginalisation is missing. Authors refer to the paper by Bäckman et al, but it would important to explain to readers why certain cut-offs (e.g., "labour market income of less than 12.5% of the median") were chosen.**

Response: Thank you for this comment. We agree these methodological choices could be made more transparent to the reader. We have therefore added the following sentences to the methods section (new text in italics):

*“In Bäckman et al.’s definition, 12.5% of the median labour market income is a pragmatic cut-off between labour market positions defined as no or negligible labour market participation and part-time or unstable work. While a full-time income at a low salary corresponds approximately to 87.5% of the median labour market income, 12.5% represents an annual income below what would be earned in 2 months in such a position.* Additionally, those with an income from disability pensions of at least 25% of the median labour market income *(which corresponds to approximately half a year of full-time disability pension)* and those registered as unemployed for at least 180 days during the year *(more than half the year)* were also included in the LMM category. *Although the precise cut-off values to a certain extent are arbitrary, these definitions have been used in several other Nordic comparative studies of labour market marginalization (31,33,34) and aligning the definitions across studies facilitates comparisons.”*

Reference 31 was already included on the list of references, while references 33 and 34 have been added:

*“33. Lorentzen T, Bäckman O, Ilmakunnas I, Kauppinen T. Pathways to adulthood: Sequences in the school-to-work transition in Finland, Norway and Sweden. Soc Indic Res. 2019;141(3):1285–305.*

*34. Jakobsen V, Korpi T, Lorentzen T. Immigration and integration policy and labour market attainment among immigrants to Scandinavia. Eur J Popul. 2019;35(2):305–28.”*

**2. Although common mental disorders typically refer to anxiety and depressive disorders, did authors consider also analysing substance abuse or psychotic disorders?**

Response: It is very probable that the trends would be different for other disorders. As our project was originally conceived (and funded!), our focus was on common mental disorders specifically, and our study is one among several using this definition (including depressive disorders and purchases of anti-depressives, anxiety disorders, obsessive-compulsive disorders, and disorders related to severe stress). Our findings have however led us to extend our focus to other disorders, such as psychotic disorders, for future papers. Care pathways are plausibly different for psychotic disorders due to severity, and we do observe higher rates of contact among young refugees for psychosis-related disorders than among their peers.

We do not find it feasible to include an analysis of psychotic disorders in the present manuscript as it, to us, represents another research question that we want to explore in its own right. But we have added the following sentence to the discussion (page 18): *“Trends may also be different for other kinds of disorders, e.g. psychotic disorders, which are less common and may entail different healthcare pathways than the disorders included in this study.”*

**3. Was there any systematic difference in results between inpatient or outpatient versus antidepressants? I would assume that antidepressant would capture also less severe cases.**

Response: This is a good point. We write in the discussion that 90% of youth with a CMD contact had purchased antidepressant medication, except for refugees in Sweden for whom it was 70% (page 17). On the one hand, this means that the results, largely, were driven by youth who had purchased antidepressant medication. On the other hand, it means that youth who had a hospital contact but had not purchased antidepressant medication were a very small sub-group. So even if they did have higher risks of marginalization (as more severe cases) and even if the pattern of utilization was different among refugees and majority youth, their contribution to the overall inequality in labor market marginalization would be small.

We have in fact checked to see whether the distribution of CMD contacts indicated through the patient registry (in and outpatient contacts) and through the pharmaceutical registry (anti-depressants) differed for refugees and majority youth. For all three variables, the original composite variable used in the manuscript, the hospital contact variable and the anti-depressants variable, the proportion with a contact was larger in the majority population than in the refugee population. We have added this information to the methods and results sections:

Methods section (page 7): *“As a sensitivity analysis, we also investigated whether contact patterns differed between the populations when considering hospital contacts (in- or outpatient) and antidepressant purchases separately.”*

Results section (page 10): “In both Denmark and Sweden, the proportion of youth with CMD was larger in the majority population than in the refugee population. *This pattern was consistent whether CMD was defined based on hospital contacts alone, purchases of antidepressants alone, or both combined (not shown). The* difference was larger among girls than boys in both countries.”

As we conclude that our registry-based CMD variable does not capture the higher mental health vulnerability of refugees as compared with their peers, it does not seem relevant to add further sub-analyses to the study as neither definition singles out refugees as particularly vulnerable. However, we do find this “non-finding” of the explanatory power of a registry-based CMD variable very relevant to the field as such variables are often used. Indeed, having a CMD-related contact (hospital or medicine) *does* have explanatory power in terms of labor market marginalization in both the refugee and the majority population; but given differences in utilization of psychiatric services, it does not contribute to explain socio-economic inequality between the two groups of young individuals, even though we know from a host of other studies that refugees are particularly vulnerable to mental distress and disorders.

**Other changes**

To align with journal requirements, we have also made the following changes to the manuscript:

- Formatting of headings
- Correction of title page formatting
- Key words removed from abstract page
- Funding and competing interests information moved
- Reference style updated
- Figure references updated
- Naming of supplementary files updated and captions added at the end of manuscript
- The ethics statement has been updated to include information about anonymization and informed consent
